# Co-Immobilization of Tri-Enzymes for the Conversion of Hydroxymethylfurfural to 2,5-Diformylfuran

**DOI:** 10.3390/molecules24203648

**Published:** 2019-10-10

**Authors:** Zhuofu Wu, Linjuan Shi, Xiaoxiao Yu, Sitong Zhang, Guang Chen

**Affiliations:** Key Laboratory of Straw Biology and Utilization, The Ministry of Education, College of Life Science, Jilin Agricultural University, Changchun 130118, China; wzf@jlau.edu.cn (Z.W.); linjuanshi84@gmail.com (L.S.); xiaoxiaoyu094@gmail.com (X.Y.); sitongzhang20@gmail.com (S.Z.)

**Keywords:** co-immobilization, hydroxymethylfurfural, 2,5-diformylfuran, galactose oxidase, catalase, horseradish peroxidase

## Abstract

Acting as a “green” manufacturing route, the enzyme toolbox made up of galactose oxidase, catalase, and horseradish peroxidase can achieve a satisfactory yield of 2,5-diformylfuran derived from 30 mM hydroxymethylfurfural. However, as the concentration of hydroxymethylfurfural increases, the substrate causes oxidative damage to the activity of the tri-enzyme system, and the accumulated hydrogen peroxide produced by galactose oxidase causes tri-enzyme inactivation. The cost of tri-enzymes is also very high. These problems prevent the utilization of this enzyme toolbox in practice. To address this, galactose oxidase, catalase, and horseradish peroxidase were co-immobilized into Cu_3_(PO_4_)_2_ nanoflowers in this study. The resulting co-immobilized tri-enzymes possessed better tolerance towards the oxidative damage caused by hydroxymethylfurfural at high concentrations, as compared to free tri-enzymes. Moreover, the 2,5-diformylfuran yield of co-immobilized tri-enzymes (95.7 ± 2.7%) was 1.06 times higher than that of separately immobilized enzymes (90.4 ± 1.9%). This result could be attributed to the boosted protective effect provided by catalase to the activity of galactose oxidase, owing to the physical proximity between them on the same support. After 30 recycles, co-immobilized tri-enzymes still achieves 86% of the initial yield. Moreover, co-immobilized tri-enzymes show enhanced thermal stability compared with free tri-enzymes. This work paves the way for the production of 2,5-diformylfuran from hydroxymethylfurfural via co-immobilized tri-enzymes.

## 1. Introduction

It is well known that hydroxymethylfurfural (HMF) is formed by the dehydration of C_6_ sugars produced from lignocellulosic biomass [1,2]. HMF was also mentioned in the US Department of Energy’s “Top 10” list of promising platform chemicals [3]. As an important oxidized derivative of HMF, 2,5-diformylfuran (DFF) has been used as the starting material for synthesizing a host of new products, such as antifungal agents [4], pharmaceuticals [5], macrocyclic ligands [6], metal-organic compounds [7], furan-containing polymers [8,9], and cross-linking agents for poly(vinyl alcohol) [10,11], hence, the selective oxidation of HMF to DFF has received increased attention recently [12].

So far, a variety of chemical oxidants have been employed for the synthesis of DFF from HMF. For instance, the application of a DMSO-potassium dichromate complex affords a 75% yield of DFF under ultrasonic irradiation at ambient temperature [13]. An oxidation reaction of HMF to DFF driven by Mn (III)–salen catalysts provides DFF at an 89% yield in pH 11.3 buffered medium at room temperature [14]. The utilization of a polymer-supported IBX amide gives DFF an 87% yield in chloroform solution [15]. The maximum observed yield of DFF is 57% with Co/Mn/Zr/Br as a catalyst at 1 bar oxygen pressure [16]. Up to 82% HMF conversion and DFF 99% selectivity was obtained by immobilized vanadyl–pyridine complexes at 130 °C [17]. The maximum HMF conversion of 84% and selectivity of 97% was obtained using vanadyl phosphate catalysts at 150 °C [18]. Cu(NO_3_)_2_/VOSO_4_ can show 99% HMF conversion and 99% DFF selectivity at 80 °C for 1.5 h [19]. Although the usage of the abovementioned chemical oxidants gives relatively satisfactory DFF yields and selectivity, their use often pollutes the environment owing to the requirement for harsh reaction conditions and the utilization of metal salts and organic solvents. Alternatively, the enzyme catalysis route requires only mild conditions and obviates the usage of toxic chemicals, and thus represents a desirabke “green” manufacturing strategy for DFF production [20,21].

**Scheme 1:**
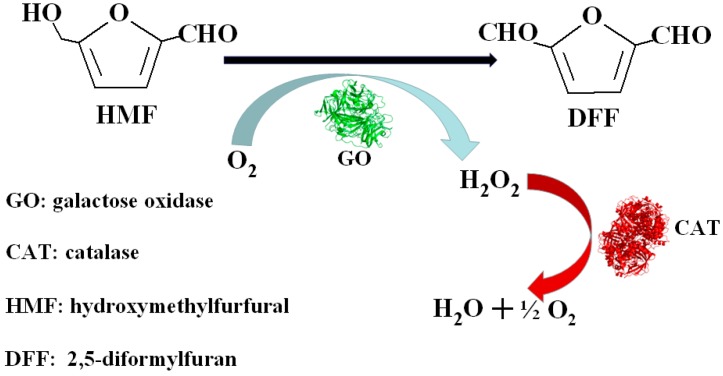
The oxidation of HMF from DFF catalyzed by enzymes.

Regrettably, studies into using enzymes as catalysts to accomplish the conversion of HMF to DFF are relatively rare. Although chloroperoxidase and hydrogen peroxide catalyze the oxidation of HMF to obtain a maximum 74% selectivity and 87% conversion to DFF, two main side products, 2,5-furandicarboxylic acid and 5-formyl-2-furancarboxylic acid, can be detected in the reaction medium, which can be obstacles to the separation of DFF [22]. The enzyme toolbox selected in this study, containing galactose oxidase, catalase, and horseradish peroxidase, offers DFF at a 91% yield and almost 100% selectivity using 30 mM HMF as feedstock in deionized water at 25 °C for 96 h [23]. Among the three enzymes, galactose oxidase plays an essential role in the oxidation of DFF [24]. Catalase decomposes H_2_O_2_ generated in the synthesis solution, providing protection for the galactose oxidase [25]. Horseradish peroxidase activates the oxidation activity of galactose oxidase by forming intramolecular crosslinks via *o,o*-dityrosine [26]. As a result, galactose oxidase acts synergistically with catalase and horseradish peroxidase in performing the oxidation of HMF to DFF (Scheme 1). However, the high cost of the three enzymes, the oxidative damage to the three enzymes caused by HMF at a high concentration [27,28], and the inactivation of the activities of the three enzymes brought about by the accumulation of hydrogen peroxide generated in the presence of a high concentration of HMF impede the large scale production of DFF.

It is well established that immobilization of enzymes is a straightforward strategy to overcome the low operational stability of enzymes, and the difficulties in their recycling [29]. In 2012, Zare et al. accidently discovered the formation of hybrid organic and inorganic nanoflowers through self-assembly of various proteins and copper phosphate crystals under mild conditions [30]. The coordination of the nitrogen atoms in the proteins and the Cu ion in the nanoflowers led to the formation of hybrid nanoflowers. As the enzyme was used as the organic component, the harvested hybrid nanoflowers take on boosted enzymatic activity and stability. The mild conditions required for preparing the hybrid nanoflowers guarantee that the conformation of the enzyme is not perturbed during self-assembly, allowing it to retain its maximum catalytic activity. The high surface area of the hybrid nanoflowers facilitates the diffusion of the substrate and product to and from the active site of the enzyme, so that it exerts enzymatic activity in the matrix. The substantial mechanical stability of the nanoflower matrix is beneficial to the utilization of the enzyme in repeated batches and in continuous operation. Therefore, this method was adopted in this work to solve the problem arising from the use of the three enzymes involved in the oxidation reaction of HMF at a high concentration.

It is the case, generally speaking, that immobilizations of multiple enzymes can be classified into two types: (1) all the enzymes are simultaneously immobilized on the same carrier, namely co-immobilization, and (2) each enzyme is independently immobilized on the carrier under their respective optimal immobilization conditions, and then the resulting separately immobilized enzymes are used together. The usage and preparation of co-immobilized enzymes are relatively complex. When all involved enzymes are immobilized on the same carrier under the identical conditions, this immobilization protocol is often non-ideal for all enzymes possessing its unique function and structure, respectively. Moreover, the difference in the stability between different enzymes raises another serious problem: as the least stable enzyme lost its activity, the half live of the whole co-immobilized multi-enzyme system shall be reduced dramatically, bringing about that the whole co-immobilized multi-enzyme system must be discarded even if the more stable enzyme in this system still maintains full activity [31]. Hence, immobilization is a non-trivial protocol for fabricating the biocatalysts. In Roberto and his colleagues’ work, the more stable enzyme was firstly immobilized on the glyoxyl support by covalent attachment, and then the least stable enzyme was immobilized on the same carrier by interfacial activation. The least stable enzyme can be released from the carrier by using detergents after its activity dramatically decreases, and then the glyoxyl support loaded with the more stable enzyme was employed to reload the fresh batch of the least stable enzyme. Using this sequential co-immobilization method, the more stable enzymes in co-immobilized multi-enzyme systems can be reused [32,33,34,35]. Although the co-immobilization of enzymes has its issues, this technique plays a critical role in certain cases in which it is necessary to achieve a rapid conversion or elimination of the reaction intermediates. As compared with separately immobilized enzymes, co-immobilized enzymes have kinetic advantages in a cascade reaction [31]. The spatial distance between the active sites of the different enzymes immobilized on the same carrier is shorter than in separately immobilized enzymes, which facilitates the efficient shuttling of the reaction intermediates in the cascade reaction, and thus brings about an improvement in reaction velocity [31,36]. These intermediates either affect the yield of the final product, or inactivate the activity of all the enzymes involved [37,38,39,40,41]. For instance, as co-immobilized tri-enzymes are comprised of glycerol dehydrogenase, NADH oxidase and catalase were employed to produce 1,3-dihydroxyacetone. The in situ decomposition of H_2_O_2_ catalyzed by catalase avoids the spontaneous oxidation of glycerol dehydrogenase and NADH oxidase, and thus markedly enhances the yield of 1,3-dihydroxyacetone [42].

Inspired by previous published work, to address problems in the oxidation of HMF, in this work enzyme co-immobilization technology was adopted because of the following reasons: (I) the enhanced rigidity of enzyme conformation via immobilization would lead to improved tolerance of the three enzymes towards the inactivation effect of HMF at a high concentration due to the multiple attachments between the enzymes and the carrier [29,43,44]; (II) the proximity effect offered by co-immobilized enzymes should be of benefit by weakening the toxic effect of hydrogen peroxide on the three enzymes during the oxidation reaction, by rapidly eliminating the hydrogen peroxide produced in the presence of a high concentration of HMF [40,41,42]; (III) the co-immobilization of enzymes would enable the reuse of the three enzymes, to reduce the cost in DFF production.

**Scheme 2:**
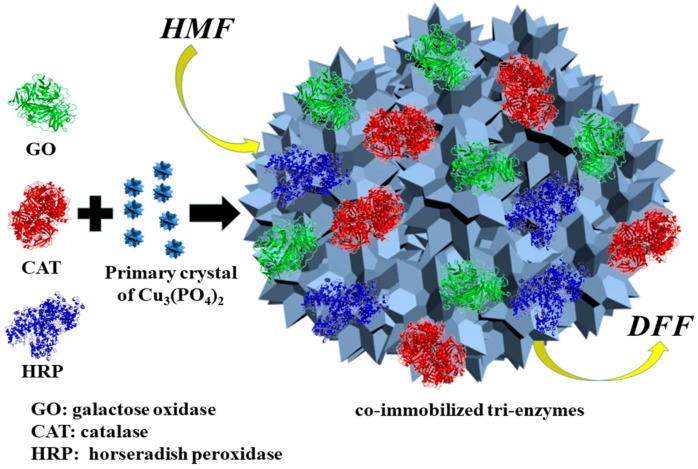
The conversion of HMF to DFF catalyzed by co-immobilized tri-enzymes.

In this study, galactose oxidase, catalase and horseradish peroxidase were co-immobilized into Cu_3_(PO_4_)_2_ nanoflower matrixes possessing perfect mechanical stability and a high surface area [30], and then the obtained co-immobilized tri-enzymes were employed in the conversion of HMF at a high concentration to DFF (Scheme 2). SEM and FTIR were employed to characterize the co-immobilized tri-enzymes. The activity and kinetic parameters of free tri-enzymes and co-immobilized tri-enzymes were assayed. The reaction conditions and the concentration of HMF, temperature and pH were optimized. A comparison between co-immobilized tri-enzymes and independently immobilized enzymes in terms of DFF yield was carried out. A comparison in thermal stability between free tri-enzymes and co-immobilized tri-enzymes was also undertaken. Then, co-immobilized tri-enzymes were conducted in successive batches. Finally, the level of H_2_O_2_ produced by free tri-enzymes, co-immobilized tri-enzymes, and separately immobilized enzymes was investigated and compared.

## 2. Results and Discussion

### 2.1. SEM Images of Co-Immobilized Tri-Enzymes

**Figure 1:**
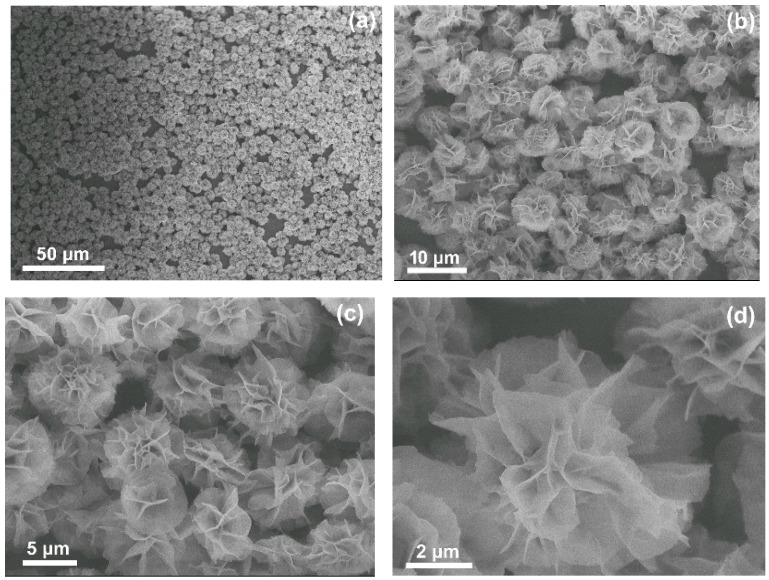
SEM images of the co-immobilized tri-enzymes with different enlargement factors (**a**–**d**).

Enzymes incorporated into nanoflowers possess nanostructures with branched morphologies, as shown in Figure 1. The average size of these nested structures is in the range of 3–8 nm. The appearance of porous flower-like structures with nanoscale feature depends on the aggregation of a number of primary nanoparticles made of copper (II) phosphorus bound by a protein’s amine groups [30]. This nanostructure, with a much higher surface area, enables the enzyme substrate to easily access its active site during the oxidation reaction.

### 2.2. FTIR Spectra of Co-Immobilized Tri-Enzymes

**Figure 2:**
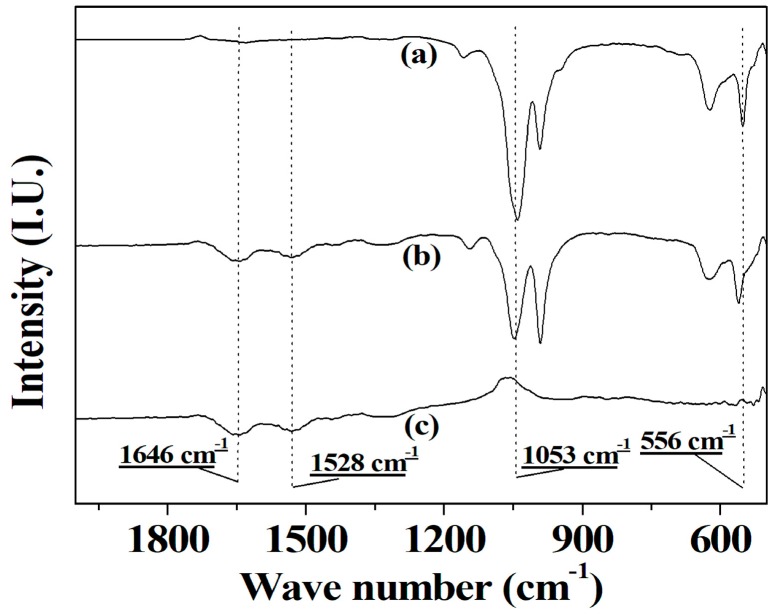
FTIR spectra of Cu_3_(PO)_4_ crystal (**a**), co-immobilized tri-enzymes (**b**) and tri-enzymes (**c**).

The FTIR spectra of enzymes, co-immobilized tri-enzymes and Cu_3_(PO)_4_ crystals are plotted in Figure 2. The spectrum of Cu_3_(PO)_4_ crystals in a curve A presents the characteristic absorption of (PO)_4_^−3^ at 1035 cm^−1^ and 556 cm^−1^ [45], and the spectrum of tri-enzymes in curve C shows the corresponding characteristic absorption of amide I and II bands of protein at 1660 cm^−1^ and 1533 cm^−1^, respectively [46]. Most importantly, the spectrum of co-immobilized tri-enzymes in curve B exhibits the characteristic absorption of both (PO)_4_^−3^ of Cu_3_(PO)_4_ crystals and amide I and II bands of protein, respectively. Hence, it is can be concluded from the presented spectra that the tri-enzymes are present in the nanoflowers.

### 2.3. Optimization of the Proportion of Enzymes

Before co-immobilization, the proportion of the three enzymes used was screened on the basis of the value of DFF yield and the level of H_2_O_2_ produced from the oxidation of HMF (μM). From Table 1, the DFF yield significantly increases with the increase of the GA/CAT/HRP ratio, ranging from 0.2/400/25 to 1.0/400/25. As the GA/CAT/HRP ratio is 1.0/400/25, the resulting co-immobilized enzymes give 64.5 ± 2.3% in DFF yield, and 4.9 ± 0.3 μM of H_2_O_2_ concomitantly formed from the oxidation of HMF could be found in the reaction mixture. With the further increase of the GA/CAT/HRP ratio, the increase of the DFF yield slows, while the level of H_2_O_2_ accumulated in the reaction medium rapidly rises. Therefore, a GA/CAT/HRP ratio of 1.0/400/25 was used in the following co-immobilization procedure.

### 2.4. Comparison of Activity

Because the self-assembly of hybrid organic–inorganic nanoflowers was conducted under mild conditions, which minimizes enzyme denaturation [30], most of the activities of the three enzymes were preserved after co-immobilization (Table 2).

### 2.5. Kinetic Parameters Analysis

Owing to the mild synthetic route for synthesizing the protein-incorporated nanoflowers [30], here the catalytic behavior of the three enzymes did not obviously change after co-immobilization or separate immobilization (Table 3).

### 2.6. Screening of Reaction Conditions

The effect of the concentration of HMF, temperature and pH on DFF yield was investigated to optimize the reaction parameters.

#### 2.6.1. Effect of HMF Concentration

**Figure 3:**
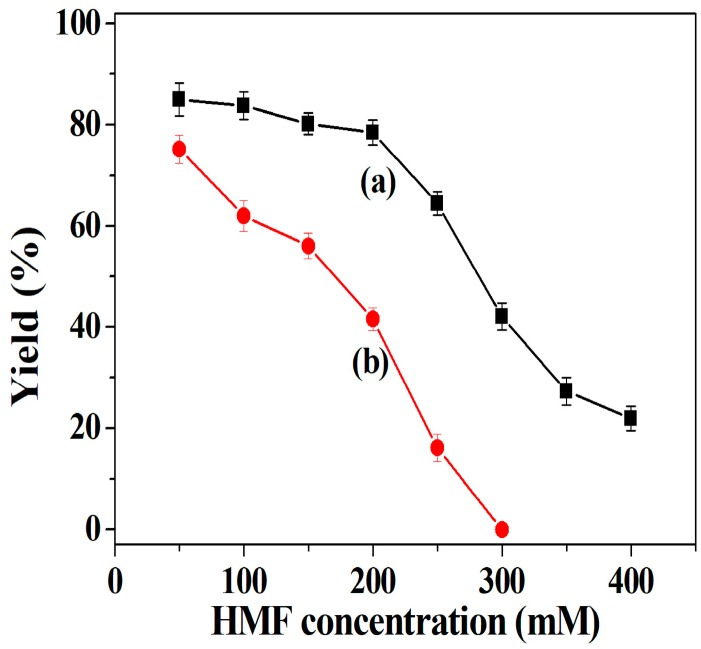
Effect of HMF concentration on DFF yield: co-immobilized tri-enzymes (**a**) and free tri-enzymes (**b**). Reaction conditions: deionized water; 25 °C; at different HMF concentrations from 50 mM to 400 mM. Free tri-enzymes (20 U of galactose oxidase, 8000 U of catalase and 500 U of horseradish peroxidase) and 35 mg of co-immobilized tri-enzymes were utilized in parallel experiments, respectively.

In practice, the oxidation reaction of HMF is implemented at a high concentration. Therefore, the effect of HMF concentration in the range of 50–400 mM on the DFF yield was surveyed. The amount of tri-enzymes loaded into the hybrid nanoflowers added to the reaction media was equal to the amount of free tri-enzymes used to evaluate whether the resistance of tri-enzymes towards the oxidative damage derived from HMF at a high concentration could be enhanced after immobilization. As for co-immobilized tri-enzymes, the DFF yield slightly decreases at a low HMF concentration ranging from 50 to 200 mM, and thereafter the DFF yield begins to drop significantly with an increasing concentration of HMF in the range of 200 to 400 mM (Figure 3a). For free tri-enzymes, DFF yield rapidly declines with the increase of the HMF concentration (Figure 3b). The results indicate that co-immobilized tri-enzymes show better tolerance towards the oxidative damage caused by HMF as compared with free tri-enzymes, suggesting that the stability of tri-enzymes is boosted via immobilization. Herein, co-immobilized enzymes still achieve a good DFF yield (78.5%) at 200 mM HMF concentration, which was employed in the subsequent study.

#### 2.6.2. Effect of pH

**Figure 4:**
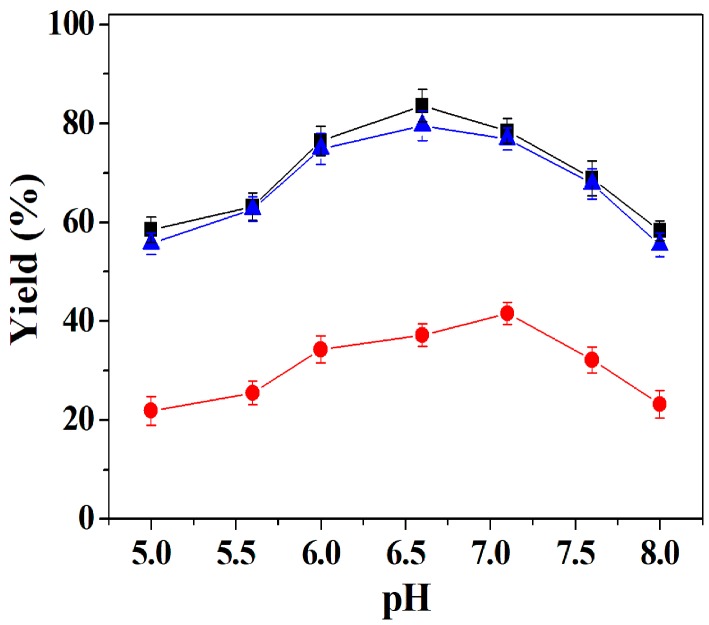
Effect of pH on the yield of DFF of co-immobilized tri-enzymes (■), separately immobilized tri-enzymes (▲), and free tri-enzymes (●). Reaction conditions: 35 mg of co-immobilized tri-enzymes or 105 mg of separately immobilized tri-enzymes (containing 35 mg of immobilized galactose oxidase, 35 mg of immobilized catalase and 35 mg of immobilized horseradish peroxidase) or free tri-enzymes (20 U of galactose oxidase, 8000 U of catalase and 500 U of horseradish peroxidase); 200 mM of HMF; 25 °C; at different pH values ranging from 5.0 to 8.0. The buffers used were citric acid–sodium citrate (pH 5.0–6.6), and Tris-HCl (pH 7.1–8.0), respectively.

Considering that a change in pH can influence the activity of enzymes, the dependence of DFF yield upon pH in the range of 5.0–8.0 was evaluated here. Figure 4 illustrates that DFF yield of co-immobilized tri-enzymes and separately immobilized tri-enzymes increased with an increasing pH value from 5.0 to 6.6, and then decreased as pH increased further. In the case of free tri-enzymes, DFF yield gradually increased with increasing pH in the range 5.5–7.1, but after 7.1 DFF yield decreased. The maximum yields of co-immobilized tri-enzymes and separately immobilized tri-enzymes were observed at pH 6.6, while free tri-enzymes showed a maximum yield at pH 7.1, which is close to the optimal pH (7.0–7.3) of galactose oxidase [47]. The tri-enzymes showed a shift in the optimal pH of 0.6 units toward the acidic region after immobilization, which may be attributed to the partitioning of protons influenced by Cu ions in the nanoflower matrix, and the mass transfer limitation of HMF [48]. In the subsequent study, oxidation reactions of co-immobilized tri-enzymes and separately immobilized tri-enzymes were carried out in Tris-HCl buffer (50 mM, pH 6.6), and for free tri-enzymes in Tris-HCl buffer (50 mM, pH 7.1), respectively.

#### 2.6.3. Effect of Temperature

**Figure 5:**
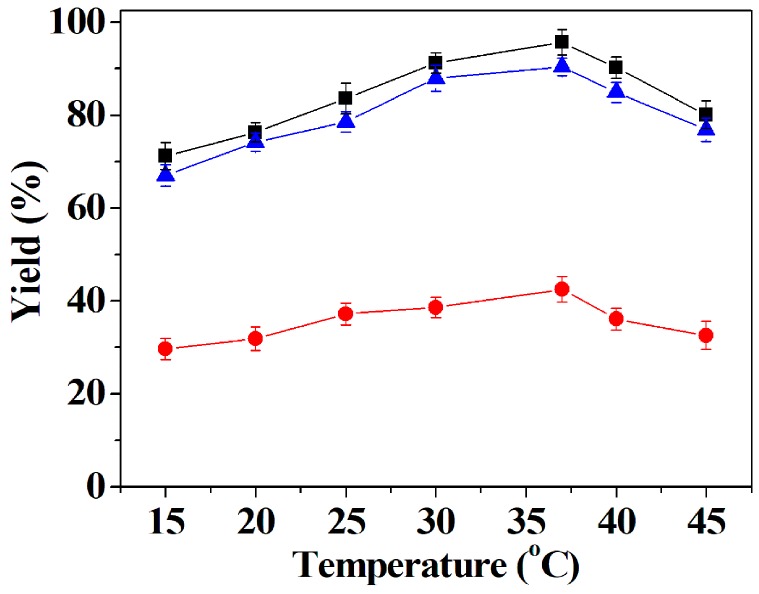
Effect of temperature on the DFF yield of co-immobilized tri-enzymes (■), separately immobilized tri-enzymes (▲) and free tri-enzymes (●). Reaction conditions: 35 mg of co-immobilized tri-enzymes or 105 mg of separately immobilized tri-enzymes (containing 35 mg of immobilized galactose oxidase, 35 mg of immobilized catalase and 35 mg of immobilized horseradish peroxidase) or free tri-enzymes (20 U of galactose oxidase, 8000 U of catalase and 500 U mg of horseradish peroxidase); 200 mM of HMF; Tris-HCl buffer (50 mM, pH 6.6) for co-immobilized tri-enzymes and separately immobilized tri-enzymes, and Tris-HCl buffer (50 mM, pH 7.1) for free tri-enzymes; at different temperatures ranging from 15 to 45 °C.

The temperature is an important parameter for the properties of enzymes [49,50,51,52]. The DFF yield was studied as a function of temperature ranging from 15–45 °C. Figure 5 shows that the DFF yield of co-immobilized tri-enzymes and separately immobilized tri-enzymes increased with the increase of the temperature between 15 and 37 °C, while DFF yield dropped with a further increase of the temperature between 37 and 45 °C. In a free tri-enzymes solution, DFF yield gradually increased at lower temperatures from 15 to 37 °C, and then it dropped significantly at higher temperatures exceeding 37 °C. Above 37 °C, in all three cases, the fall in the DFF yield can be ascribed to the inactivation of the enzymes. The results signify that the optimal yield of co-immobilized tri-enzymes, separately immobilized tri-enzymes, and free tri-enzymes occurred at 37 °C, which was adopted in the following study.

### 2.7. Thermal Stability

**Figure 6:**
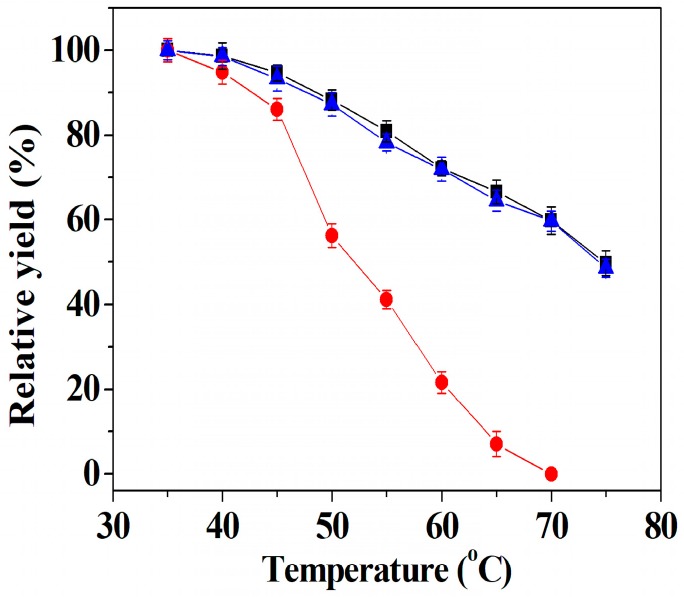
The effect of heat incubation on the DFF yield of co-immobilized tri-enzymes (■), separately immobilized tri-enzymes (▲) and free tri-enzymes (●). Reaction conditions: 35 mg of co-immobilized tri-enzymes or 105 mg of separately immobilized tri-enzymes (containing 35 mg of immobilized galactose oxidase, 35 mg of immobilized catalase and 35 mg of immobilized horseradish peroxidase) or free tri-enzymes (20 U of galactose oxidase, 8000 U of catalase and 500 U of horseradish peroxidase); 200 mM of HMF; Tris-HCl buffer (50 mM, pH 6.6) for co-immobilized tri-enzymes and separately immobilized tri-enzymes, and Tris-HCl buffer (50 mM, pH 7.1) for free tri-enzymes; at 37 °C.

To compare the difference in structural stability among free tri-enzymes, co-immobilized tri-enzymes, and separately immobilized tri-enzymes, heat inactivation experiments were conducted in the temperature range of 35–75 °C. Figure 6 shows that although the relative DFF yields of the three samples declined with the increasing treatment temperature, the decline rates of the relative DFF yields of co-immobilized tri-enzymes and separately immobilized tri-enzymes were much slower than that of the free tri-enzymes. The results also indicate that the relative DFF yield of free tri-enzymes is zero percent beyond 70 °C, while co-immobilized tri-enzymes afford 59.8 ± 3.2% and 49.8 ± 2.9% of relative DFF yield at 70 and 75 °C, respectively, and separately immobilized tri-enzymes exhibit 59.7 ± 2.4% and 48.6 ± 2.1% of relative DFF yield at 70 and 75 °C, respectively. The substantial improvement in the thermal stability of co-immobilized tri-enzymes and separately immobilized tri-enzymes can be ascribed to the enhancement of the rigidification of the tri-enzymes’ tertiary structure, caused by the coordination bond between the amide groups in the tri-enzyme backbone and Cu ions in the hybrid nanoflowers [30,53]. The results also suggest that there is no significant difference in thermal stability between tri-enzymes co-immobilized into the nanoflowers and tri-enzymes separately immobilized into the nanoflowers.

### 2.8. Comparison of DFF Yield

The data in Table 4 confirm that co-immobilized tri-enzymes achieve an increase in DFF yield of 106% compared with separately immobilized enzymes. The free tri-enymes exhibit a low DFF yield due to the inactivation effect of HMF on its activities. One of the primary goals in the co-immobilization of different enzymes is to diminish deleterious side products [54]. The enhancement in DFF yield should be attributed to the favorable proximity effect of co-immobilized tri-enzymes, which benefits catalase by instantaneously removing H_2_O_2_ generated by galactose oxidase on the same matrix during HMF oxidation. In Guisan and his co-workers’ report, glycerol dehydrogenase, NADH oxidase and catalase were co-immobilized on agarose beads to accomplish the selective oxidation of glycerol to 1,3-dihydroxyacetone, and the co-immobilized tri-enzyme system achieved an increase in the product yield of 190% compared with a separately immobilized tri-enzyme system, owing to in situ elimination of H_2_O_2_ [42], which is in accordance with our results. Cu_3_(PO)_4_ crystals have no catalytic activity for the oxidation of HMF.

### 2.9. Reusability of Co-Immobilized Tri-Enzymes

**Figure 7:**
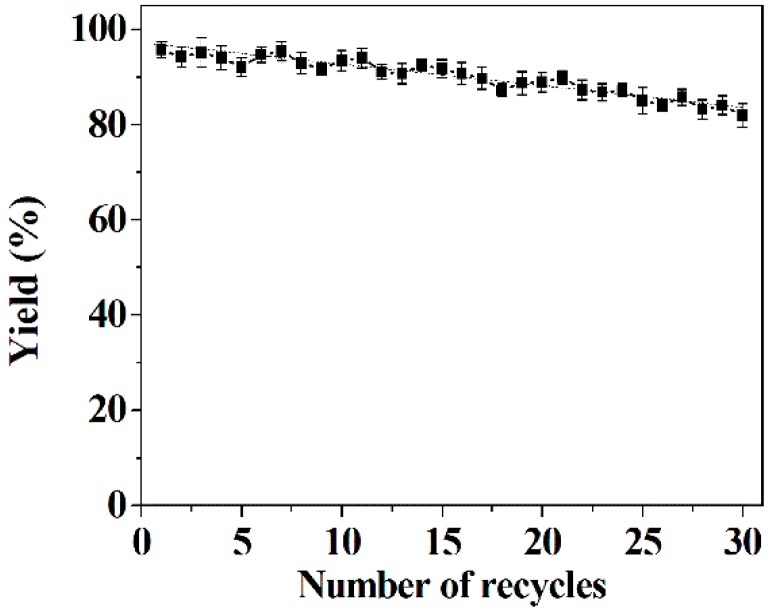
Reusability of co-immobilized tri-enzymes. Reaction conditions of each run: 35 mg of co-immobilized tri-enzymes; 200 mM of HMF; 37 °C; Tris-HCl buffer (50 mM, pH 6.6).

One of the primary advantages of the utilization of immobilization enzyme techniques is the potential for reuse of the enzyme [55,56,57,58]. The operational stability of co-immobilized tri-enzymes is illustrated in Figure 7. The co-immobilized tri-enzymes still maintain 86% of the initial yield, even after 30 runs. After each run, the reaction system was centrifuged, and no absorbance at OD_280_ nm in the resulting suspension was detected by UV-vis spectrophotometer, confirming that no leakage of enzymes in the nanoflower matrix occurred. Hence, the repeated reaction, separation and rinsing steps in successive reactions may give rise to a slight loss of DFF yield. The outstanding reusability can be attributed to: (1) the stable conjunction between the tri-enzymes and the hybrid nanoflowers via the coordinate bond between the three enzymes and Cu ions in the nanoflowers, (2) the enhanced rigidification of the tri-enzymes’ tertiary structure by immobilization, and (3) the remarkable mechanical stability of the Cu_3_(PO_4_)_2_ nanoflower matrix, which was demonstrated by our previous works in which lipase-incorporated nanoflowers retained 98.7% of the initial enzyme activity and 95.6% of the initial *E* value after 10 continuous batches for resolution of (*R*,*S*)-2-pentanol and laccase, and in other nanoflowers 93.2% of the initial activity for synthesizing viniferin was maintained after 10 consecutive runs [59,60]. The perfect reusability of co-immobilized tri-enzymes permits their industrial amplification potential for producing DFF.

### 2.10. Determination of the Level of H_2_O_2_ in Different Reaction Systems

The level of hydrogen peroxide released during the enzymatic process was measured with soluble, either immobilized separately or co-immobilized, biocatalysts. The results confirm that 2.5 ± 0.4 μm of H_2_O_2_ could be detected in the reaction solution when the co-immobilized tri-enzymes were used as catalysts, while 27.2 ± 2.2 μm of H_2_O_2_ was found in the reaction mixture when the independently immobilized tri-enzymes were employed, and 253.3 ± 3.7 μm of H_2_O_2_ was present in the reaction system catalyzed by free tri-enzymes (Table 5). Guisan et al. demonstrated that a co-immobilized three enzymes system made up of glycerol dehydrogenase, NADH oxidase, and catalase exhibited a 115% increase in efficiency of H_2_O_2_ elimination in comparison with separately immobilized enzymes, which is in agreement with our results [42]. The higher H_2_O_2_ removal capability of co-immobilized tri-enzymes compared with independently immobilized tri-enzymes confirms the existence of a proximity effect between the galactose oxidase and catalase on the same carrier. The lower H_2_O_2_ removal capability of free tri-enzymes as compared with other catalysts is due to the inactive effect of HMF at a higher concentration on the activity of free catalase.

## 3. Materials and Methods

### 3.1. Materials

Galactose oxidase from *Dactylium dendroides* (500–1500 U/mg protein, one unit generates a ΔA425 of 1.0 per minute at pH 6.0 at 25 °C in a peroxidase and o-tolidine system), catalase from bovine liver (2000–5000 U/mg protein, one unit decomposes to 1.0 micromole of hydrogen peroxide per minute at pH 7.0 at 25 °C), horseradish peroxidase (>200 U/mg protein, one unit offers 1.0 mg purpurogallin from pyrogallolin after 20 sec at pH 6.0 at 20 °C), HMF, KBr, bovine serum albumin (BSA), choline chloride, and glycerol were obtained from Sigma-Aldrich Chemical Co. (St. Louis, MO, USA). 3-Methoxybenzyl alcohol, hydrogen peroxide solution (30 wt % in H_2_O), pyrogallol, hydroxymethylfurfural (HMF), 2,5-diformylfuran (DFF), and SIGMAFAST DAB with Metal Enhancer Tablet Sets (DAB) were also supplied by Sigma-Aldrich Chemical Co. All chemicals and reagents were of analytical grade. All aqueous solutions were prepared with Milli-Q water.

### 3.2. Co-Immobilization of Tri-Enzymes

The co-immobilization procedure of tri-enzymes is similar to the one described in previous work [30]. Taking in account that the best DFF yield could be obtained when using soluble galactose oxidase (10 U), catalase (4000 U) and horseradish peroxidase (250 U) as catalysts in deionized water [23], the three enzymes were added to the immobilization system according to the abovementioned ratio. In brief, 650 U of galactose oxidase, 260 KU of catalase and 16.25 KU of horseradish peroxidase were dissolved in 3 L of PBS buffer (50 mmol/L, pH 7.4), before 20 mL of CuSO_4_ (120 mM) was added. The proportion of the three enzymes in the co-immobilized preparation was 0.01:1.1:1.3. The mixture was incubated at 4 °C for 3 days. Then, the blue product was harvested by centrifugation at 3500× *g* for 20 min, and then rinsed with deionized water. Finally, the product was dried at room temperature. The Bradford protein assay was used to determine the protein concentration in the supernatant and washing solutions, and BSA was used as standard [61]. The immobilization yields (%) of the tri-enzymes and separately immobilized enzymes were calculated by the formula below:

Y_1_ = ((W_1_ − W_2_)/W_1_) × 100 (1)

where Y_1_ = encapsulation yield (%), W_1_ = the amount of the enzymes employed in immobilization system, and W_2_ = the amount of the enzymes present in the supernatant and washing solutions. Herein, the immobilization yield of the tri-enzymes was 96%.

The procedures for the independently immobilized enzymes were the same as for the co-immobilization route, except that only one enzyme and BSA, which was employed to replace the two other enzymes, were co-immobilized into the nanoflowers. The immobilization yield of separately immobilized enzymes was also 96%.

### 3.3. Characteristics of Co-Immobilized Tri-Enzymes

Scanning electron microscopy (SEM) images were taken using a JSM-6700F electron microscope (JEOL, Tokyo, Japan) operated at 30 kV. Fourier-transform infrared spectroscopy (FTIR) spectra were recorded on a 5700 FTIR spectrometer (Nicolet, Madison, WI, USA) using KBr pellets at a resolution of 4 cm^−1^.

### 3.4. Enzyme Assay

#### 3.4.1. Galactose Oxidase

The activities of free galactose oxidase, co-immobilized galactose oxidase, and independently immobilized galactose oxidase were measured using 3-methoxybenzyl alcohol as the substrate [26]. The oxidation of 3-methoxybenzyl alcohol was triggered by the addition of a suitable amount of catalyst sample (50 mg of co-immobilized galactose oxidase, 50 mg of independently immobilized galactose oxidase, or 100 μL of 0.3 mg/mL free galactose oxidase solution prepared by Tris-HCL (50 mM, pH 7.1)) to 10 mL of 0.06 M 3-methoxybenzyl alcohol dissolved in the same buffer. The reaction systems were incubated at 37 °C for 3 min. For co-immobilized galactose oxidase and independently immobilized galactose oxidase, the aliquots of the reaction mixture (1 mL) were taken every 30 s and then filtrated by a membrane filter (0.22 µm pore size). The absorbance increase of the filtrate at 314 nm (ε = 2691 M^−l^ cm^−1^) was recorded using an 8453 UV-VIS spectrophotometer (Agilent, Santa Clara, CA, USA). The determination of the activity of free enzymes followed the same procedure, except that the filtration step was omitted. One unit was defined as the amount of enzyme that formed 1.0 µ mol of aldehyde per minute per milligram of galactose oxidase located in the hybrid nanoflowers, or free galactose oxidase under the conditions described.

#### 3.4.2. Catalase

The activities of free catalase, co-immobilized catalase, and independently immobilized catalase were determined by following the decomposition of H_2_O_2_ [62]. The hybrid nanoflowers containing enzymes (5 mg) were suspended in 50 mL of deionized water and allowed to stand for 5 min. A suitable amount of catalyst sample (100 µL of 0.1 mg/mL co-immobilized catalase, 100 µL of 0.1 mg/mL independently immobilized catalase, or 100 µL of 0.01 mg/mL free catalase) was added to 10 mL of 12 mM H_2_O_2_ to start the decomposition reaction. The separation of co-immobilized catalase and independently immobilized catalase from the reaction media was carried out by the filtration steps described above. The absorbance decrease of filtrate at 240 nm (ε = 43.6 M^−1^ cm^−1^) was monitored using the Agilent 8453 UV-VIS spectrophotometer. The determination of the activity of free enzymes followed the same procedure, but without the filtration step. One unit was defined as the amount of enzyme that decomposed 1.0 µ mol of H_2_O_2_ per minute per milligram of catalase encapsulated into the hybrid nanoflowers, or free catalase under the conditions described.

#### 3.4.3. Horseradish Peroxidase

The activities of free horseradish peroxidase, co-immobilized horseradish peroxidase, and independently immobilized horseradish peroxidase were assayed using pyrogallol and H_2_O_2_ as substrates [63]. The reaction mixture contained: 100 mM phosphate buffer, pH 6.0, 10 mM H_2_O_2_ and 13 mM pyrogallol, and a suitable amount of catalyst sample (100 µL of 0.1 mg/mL co-immobilized horseradish peroxidase, 100 µL of 0.1 mg/mL independently immobilized horseradish peroxidase or 100 µL of 0.01 mg/mL free horseradish peroxidase) in a final volume of 20 mL. The reaction mixture was incubated at 30 °C for 3 min. The absorbance increase at 430 nm (ε = 2.47 mM^−l^ cm^−1^) was detected using the Agilent 8453 UV-VIS spectrophotometer. As mentioned above, co-immobilized horseradish peroxidase and independently immobilized horseradish peroxidase were separated by a filtration step to complete the UV-VIS detection. One unit was defined as the amount of enzyme that produced 1.0 µ mol of purpurogallin per minute per milligram of horseradish peroxidase encapsulated into the hybrid nanoflowers, or free horseradish peroxidase.

### 3.5. Kinetic Analysis

Comparison of the Michaelis–Menten constants *K*m and *V*max of the soluble enzymes, the enzymes in co-immobilized tri-enzymes, and independently immobilized enzymes were implemented. The effect of substrate concentration on the initial rates of different catalysts was determined via the spectrophotometric method using the assays described in Section 3.4. As for galactose oxidase, the concentration of 3-methoxybenzyl alcohol in Tris-HCL (50 mM, pH 7.1) was varied from 3 to 60 mM. For catalase, the concentration of H_2_O_2_ was varied from 5 to 60 mM. For horseradish peroxidase, the concentration of pyrogallol in 100 mM phosphate buffer was varied from 1 to 8 mM. After acquiring the initial rates of different catalysts versus their substrate concentration data, the values of *V*max and *K*m could be calculated through non-linear regression using the Origin software 8.5 software (OriginLab, Northampton, MA, USA).

### 3.6. Oxidation of HMF by Co-Immobilized Tri-Enzymes

The oxidation reaction of HMF was conducted by mixing 35 mg of co-immobilized tri-enzymes and 5 mL of 200 mM HMF prepared by 50 mM Tris-HCl buffer (pH 6.6) in a vessel covered to prevent evaporation. The resulting mixture was consecutively stirred in a shaking bath at 160 rpm at 37 °C for 7 days. The resulting mixture was treated by air bubbling for 15 min each day. After centrifugation at 3500× *g* for 5 min, 5 mL of ethyl acetate was added to the supernatant. The obtained mixture was extracted using 5 mL of deep eutectic solvent (choline chloride: glycerol (1:2, mol/mol)) three times. The obtained mixture stood for 10 min at 25 °C to form a biphasic system. The HMF and DFF could be found in the upper and lower phases, respectively. Their amounts were determined by HPLC analysis. The DFF yield was calculated according to the following formula:

Y_2_ = (W_3_/W_4_) × 100 (2)

where Y_2_ = DFF yield (%), W_3_ = the amount of DFF, and W_4_ = the amount of HMF.

### 3.7. HPLC Analysis

Analyses of HMF and DFF were implemented by RP-HPLC using Waters 1525 Binary Pumps and a Waters 2489 UV-Visible detector (Waters, Milford, MA, USA) on a Zorbax SB C-18 column (250 mm × 4.6 mm, 5 μm, Agilent) The mobile phase consisted of acetonitrile and 0.1 wt% trifluoroacetic acid aqueous solution (*v*/*v* = 15:85) at 0.8 mL/min. The column oven temperature was maintained at 35 °C. The UV detection wavelength was 284 nm. Under these conditions, the retention times of HMF and DFF were 5.1 and 6.9 min, respectively.

### 3.8. Thermal Stability

Suitable amounts of soluble tri-enzymes were dissolved in sodium Tris-HCL buffer (50 mM, pH 7.1) and co-immobilized tri-enzymes and separately immobilized tri-enzymes were dissolved in sodium Tris-HCL buffer (50 mM, pH 6.6), respectively, and then the resulting mixtures were incubated at different temperatures ranging from 35 to 75 °C for 2 h. Then, the treated tri-enzymes were assessed in terms of the oxidation of HMF. The DFF yields of soluble tri-enzymes, separately immobilized tri-enzymes, and co-immobilized tri-enzymes were examined to assess their thermal stability. The relative yield (%) was calculated by the ratio of the residual DFF yield to the initial DFF yield of each sample.

### 3.9. Reusability

After each cycle, co-immobilized tri-enzymes were separated from the reaction mixture by centrifugation at 3500× *g* for 3 min, washed with deionized water three times, and dried at 65 °C in a vacuum oven. The resulting powder was used in the next run under the same conditions. The residual DFF yield of the recycled co-immobilized tri-enzymes was calculated each run by considering the initial DFF yield to be 100%.

### 3.10. Determination of the Level of H_2_O_2_ Produced during the Oxidation Reaction

The DAB/HRP method with slight modification was adopted to measure the quantification of released H_2_O_2_ in the reaction mixture [64]. During the oxidation reaction, the aliquots of the reaction mixture (1 mL) were taken every 1 day, and then filtrated by a membrane filter (0.22 µm pore size). A suitable amount of DAB and HRP was added to the filtrate, and then the absorbance increase of filtrate at 460 nm was recorded by an Agilent 8453 UV-VIS spectrophotometer. The absorbance at 460 nm was compared to a standard curve, which was prepared separately using different known concentrations of H_2_O_2_. The H_2_O_2_ concentration value was the mean for data obtained during the oxidation reaction.

### 3.11. Statistical Analysis

The data expressed in various studies were plotted using Origin 8.5 and expressed as the standard error (±). Each value represents the mean for three independent experiments.

## 4. Conclusions

In summary, galactose oxidase, catalase and horseradish peroxidase were immobilized into Cu_3_(PO_4_)_2_ nanoflowers to resist the oxidative damage aroused by the hydroxymethylfurfural substrate at a high concentration. Although these three enzymes after co-immobilization do not have advantages in activity and kinetic parameters in their model substrate assay compared with their free counterparts, co-immobilized tri-enzymes manifest a substantial improvement in tolerance towards the inactivation effect caused by hydroxymethylfurfural at a high concentration. The 2,5-diformylfuran yield of co-immobilized tri-enzymes is superior to that of free tri-enzymes after incubating at different temperatures ranging from 35 to 75 °C, confirming the remarkably enhanced thermal stability of co-immobilized tri-enzymes. Moreover, co-immobilized tri-enzymes show better performance in the conversion of hydroxymethylfurfural to 2,5-diformylfuran as compared with independently immobilized enzymes, demonstrating the role of physical proximity of galactose oxidase and catalase on the same support. After 30 cycles, co-immobilized tri-enzymes still retain 86% of the initial yield. A comparison in the level of released H_2_O_2_ revealed that the physical proximity effect of active sites of different enzymes on the same carrier is important. This is beneficial to the combination comprised of these three enzymes to exert their catalytic activity, even in the presence of 200 mM hydroxymethylfurfural. A large number of chemical catalysts have been employed to fulfill the conversion of lignocellulosic biomass to hydroxymethylfurfural. We predict that the combination of chemical catalysts and co-immobilized tri-enzymes explored in this paper would enable the conversion of lignocellulosic biomass to 2,5-diformylfuran. The superior stability and perfect reusability of co-immobilized tri-enzymes would allow us to further explore its potential in lignocellulosic biomass conversion.

## Figures and Tables

**Table 1:** The optimization of the proportion of enzymes added to the co-immobilization system.

Unit Ratio of GA/CAT/HRP Used ^a^	Yield (%)	Concentration of H_2_O_2_ Produced from the Oxidation of HMF (μm)
0.2/400/25	44.8 ± 1.8	ND
0.5/400/25	56.2 ± 2.8	ND
1.0/400/25	64.5 ± 2.3	4.9 ± 0.3
2.0/400/25	66.1 ± 2.2	40.7 ± 0.3
4.0/400/25	66.4 ± 1.9	92.9 ± 0.4

^a^ Reaction conditions: deionized water; 25 °C; at 250 mM HMF.

**Table 2:** Comparison of activity of free enzymes, co-immobilized enzymes, and independently immobilized enzymes.

Samples	Activity ^a^ (U mg^−1^)
Free galactose oxidase	166.1 ± 3.2
Galactose oxidase in co-immobilized tri-enzymes	159.2 ± 2.7
Independently immobilized galactose oxidase	157.4 ± 2.1
Free catalase	2270.5 ± 29.4
Catalase in co-immobilized tri-enzymes	2245.3 ± 24.3
Independently immobilized catalase	2258.6 ± 28.7
Free horseradish peroxidase	3658.2 ± 34.2
Horseradish peroxidase in co-immobilized tri-enzymes	3583.9 ± 42.3
Independently immobilized horseradish peroxidase	3547.7 ± 36.1

^a^ Activity refers to the amount of product formed per minute per milligram of protein.

**Table 3:** Comparison of the kinetic parameters of free enzymes, co-immobilized enzymes, and independently immobilized enzymes.

Parameter	*K*m (mM)	*V*max (µmol min^−1^ mg^−1^)
Free galactose oxidase	17.6 ± 1.3	216.4 ± 5.7
Galactose oxidase in co-immobilized tri-enzymes	17.8 ± 1.6	207.3 ± 3.1
Independently immobilized galactose oxidase	17.4 ± 1.7	205.6 ± 4.7
Free catalase	26.9 ± 0.1	7218.6 ± 26.4
Catalase in co-immobilized tri-enzymes	25.2± 0.1	7015.4 ± 26.6
Independently immobilized catalase	26.3 ± 0.5	7183.8 ± 89.5
Free horseradish peroxidase	2.21 ± 0.04	4377.7 ± 28.4
Horseradish peroxidase in co-immobilized tri-enzymes	2.12 ± 0.03	4356.8 ± 25.2
Independently immobilized horseradish peroxidase	2.15 ± 0.06	4334.2 ± 31.8

**Table 4:** Comparison of the DFF yield of co-immobilized enzymes and independently immobilized enzymes.

Samples	Yield (%)
Co-immobilized tri-enzymes ^a^	95.7 ± 2.7
Immobilized enzymes independently ^b^	90.4 ± 1.9
Free tri-enzymes ^c^	53.8 ± 2.3
The nanoflower matrix	ND

^a^ Reaction conditions: 35 mg of co-immobilized tri-enzymes; 200 mM of HMF; 37 °C; Tris-HCl buffer (50 mM, pH 6.6). ^b^ Reaction conditions: 35 mg of immobilized galactose oxidase, 35 mg of immobilized catalase and 35 mg of immobilized horseradish peroxidase; 200 mM of HMF; 37 °C; Tris-HCl buffer (50 mM, pH 6.6). ^c^ Reaction conditions: Free tri-enzymes (20 U of galactose oxidase, 8000 U of catalase and 500 U of horseradish peroxidase); 200 mM of HMF; 37 °C; Tris-HCl buffer (50 mM, pH 6.6).

**Table 5:** Comparison of the level of H_2_O_2_ produced during the oxidation of HMF catalyzed by free galactose oxidase, independently immobilized galactose oxidase, and co-immobilized galactose oxidase.

Catalysts	The Concentration of H_2_O_2_ Produced from the Oxidation of HMF (μm)
Free tri-enzymes	253.3 ± 3.7
Independently immobilized tri-enzymes	27.2 ± 2.2
Co-immobilized tri-enzymes	2.5 ± 0.4
